# Gauging food and nutritional care quality in hospitals

**DOI:** 10.1186/1475-2891-11-66

**Published:** 2012-09-06

**Authors:** Rosa Wanda Diez-Garcia, Anete Araújo de Sousa, Rossana Pacheco da Costa Proença, Vania Aparecida Leandro-Merhi, Edson Zangiacomi Martinez

**Affiliations:** 1 Laboratory of Food Practices and Behavior – PrátiCA, Nutrition and Metabolism, Department of Internal Medicine, Faculty of Medicine of Ribeirão Preto, University of São Paulo, Av. Bandeirantes, 3900, Ribeirão Preto, SP 14049-900, Brazil; 2 Department of Nutrition, Federal University of Santa Catarina, Campus Universitário, Florianópolis, SC, 88040-900, Brazil; 3 Faculty of Nutrition, PUC Campinas, Av. John Boyd Dunlop, s/n., Campinas, SP, 13060-904, Brazil; 4 Department of Social Medicine, Faculty of Medicine of Ribeirão Preto, University of São Paulo, Av. Bandeirantes, 3900, Ribeirão Preto, SP, 14049-900, Brazil

**Keywords:** Food care, Nutritional care, Public hospital, Private hospital

## Abstract

**Background:**

Food and nutritional care quality must be assessed and scored, so as to improve health institution efficacy. This study aimed to detect and compare actions related to food and nutritional care quality in public and private hospitals.

**Methods:**

Investigation of the Hospital Food and Nutrition Service (HFNS) of 37 hospitals by means of structured interviews assessing two quality control corpora, namely nutritional care quality (NCQ) and hospital food service quality (FSQ). HFNS was also evaluated with respect to human resources per hospital bed and per produced meal.

**Results:**

Comparison between public and private institutions revealed that there was a statistically significant difference between the number of hospital beds per HFNS staff member (p = 0.02) and per dietitian (p < 0.01). The mean compliance with NCQ criteria in public and private institutions was 51.8% and 41.6%, respectively. The percentage of public and private health institutions in conformity with FSQ criteria was 42.4% and 49.1%, respectively. Most of the actions comprising each corpus, NCQ and FSQ, varied considerably between the two types of institution. NCQ was positively influenced by hospital type (general) and presence of a clinical dietitian. FSQ was affected by institution size: large and medium-sized hospitals were significantly better than small ones.

**Conclusions:**

Food and nutritional care in hospital is still incipient, and actions concerning both nutritional care and food service take place on an irregular basis. It is clear that the design of food and nutritional care in hospital indicators is mandatory, and that guidelines for the development of actions as well as qualification and assessment of nutritional care are urgent.

## Background

Changes in population age distribution and lifestyle require that these institutions undergo modifications, in order to meet the current demands of society such as increased life expectancy and new disease profiles [1-4]. As part of the national health systems, these changes had an impact on the hospitals. The emergence of novel technologies, the constant need for recycling of knowledge and abilities due to reformulations of scientific paradigms, and the hospital costs implied in this recycling justify the need for assessment of quality and efficacy in this health segment [5].

Nutritional status in hospital inpatient has been the object of many studies [6-11], but there are only a few literature works on quality indicators concerning hospital food and nutrition services (HFNSs) as well as food and nutritional care actions conducted by dietitians in health institutions [12-15]. Hospital expectations often place the food and nutrition service as an undervalued support service [16], even though changes and improvements in hospital diets and nutritional care can prevent nutritional aggravations [7,8,12,17] that have a negative impact on the length of hospital stay and hospitalization costs [10,11,18-20]. Moreover, such amelioration can also impact patient’s perception of the hospitalization experience positively [21,22].

European research on food and nutritional care in hospital has recognized that it is necessary to define responsibilities, promote staff qualification, enable patients’ participation in nutritional decisions, and integrate the health care team into nutritional care [14]. A comparative study on nutritional care management involving two hospital institutions, namely a French hospital and a Brazilian hospital, has detected fragmentation of dietitians’ actions due to existence of different interlocutors and to the unpredictability inherent to food preparation procedures [23].

Food and nutritional care actions regarding both patient assistance and food quality must be well delineated and become an effective practice in health institutions. Different management sectors, namely the clinical and administrative areas, should be involved in the process, and actions should be recognized and evaluated by regulating agencies concerned with hospital quality control.

Standardization of nutritional care practice in the United States was implemented by the American Dietetic Association (ADA) in 1987. In 1993, the Clinical Nutrition Management executive committee selected professionals who later developed and assessed the standard nutritional practices that should be employed for management of the clinical nutritional area, which gave rise to a standard reference for this field [24]. Knowledge about the actual state of food and nutritional care is a crucial stage for delivery of good quality hospital assistance. This kind of diagnosis enables future comparisons and allows for evaluation of implemented changes, which is essential for action planning [25].

This study aimed to detect and compare actions relative to food and nutritional care quality in hospital in public and private hospitals.

## Methods

Thirty-seven hospitals were investigated. Twenty-seven hospitals were located in municipalities of the state of São Paulo, more specifically Campinas and Ribeirão Preto, whereas ten hospitals were located in the city of Florianópolis, capital of the state of Santa Catarina. Both Campinas and Ribeirão Preto are important scientific-technological health centers with various services of nationwide recognition. The cities included in the present work are home to various public (federal and state) and private universities. Campinas, Ribeirão Preto, and Florianópolis have an estimated population of 1,039,297; 547,417; and 396,723 inhabitants, respectively.

In the states of São Paulo and Santa Catarina there are 2.29 (0.58 public and 1.71 private) and 2.66 (0.67 public and 1.99 private) hospital beds/ 1,000 inhabitants, respectively. The metropolitan region of Florianópolis holds a larger concentration (72.7%) of public beds (2.61 of the 3.59 hospital beds/1,000 inhabitants are public), while the metropolitan region of Campinas has a larger proportion (74.6%) of private beds (1.47 of the 1.97 hospital beds/1,000 inhabitants are private) [26].

The following inclusion criteria were considered: hospitals located in the studied municipalities, regardless of the type – general, specialized, public, private, small (up to 50 beds), medium-sized (between 51 and 150 beds), or large (more than 150 beds) – that agreed to take part in the research. Psychiatric hospitals, day hospitals, nursing homes, and shelters were not included. Hospitals that did not have a dietitian responsible for the Hospital Food and Nutrition Service (HFNS) were excluded because this is compulsory according to the Brazilian law and hospitals that refused to participate in the study were not included, either. Although the healthcare team is responsible for nutritional screening and other actions, the piece of this investigation was to evaluate the dietitians actions of the hospitals food and nutritional services. An informed consent for the participation in the research was obtained from both the interviewee and a representative of the participating institution. The project was approved by the Research Ethics Committee of Faculdade de Medicina de Ribeirão Preto, University of São Paulo.

Data was collected by means of a structured interview, using a questionnaire designated Instrument for Evaluation of Food and Nutritional Care in Hospital – IEFNCH [27]. This questionnaire contained open and closed questions that helped diagnose hospital activities supporting food and nutritional care. The questions were directed to the hospital nutrition service coordinators during two visits that had been pre-arranged with the institution. The present IEFNCH was based on instruments published in similar works [13-15,24,28-30]. After the application of IEFNCH, a data bank containing information about various aspects of the institutions under study was built. We organized these data as criteria, according to these references used to qualify this instrument [27]. The size of the HFNS was measured by the number of hospital beds per dietitian, the number of HFNS staff members (kitchen staff) per dietitian, the number of produced meals per HFNS staff member, and the number of produced meals per dietitian. In these cases all dietitians that worked in these hospitals were considered, which include, clinical dietitian, dietitian who works in the management of meal production and the dietitian coordinator, if there was one.

Activities carried out by the HFNS were divided into two corpora of criteria, one relative to nutritional care quality (NCQ) and another concerning quality of the actions related to hospital food service (FSQ). Descriptive statistics was accomplished for the NCQ and FSQ criteria in terms of the legal nature and location of the institution. Differences in the criteria between public and private hospitals were considered substantial, moderate, and small when they were equal to 30%, less than 30 to 15%, and less than 15%, respectively (Table 1).

**Table 1:** Criteria comprising the nutritional care quality (NCQ) and food service quality (FSQ) indicators

**NCQ INDICATORS (100%)**	**Employed Criteria**
**INPATIENT DIETARY COVERAGE ACTIONS (A) (25%)**	A1.Duty shift system in the area of clinical nutrition
	A2.Supervision of meal distribution in the ward
	A3.Routine visits to patients
**EVALUATION AND MONITORING OF NUTRITIONAL STATUS ACTIONS (B) (25%)**	B1.Nutritional status evaluation (complete)
	B2.Nutritional status monitoring
	B3.Entry of nutritional care information in the medical record
	B4.Filling in forms about nutritional care
	B5.Nutritional guidance at discharge
	B6.Assistance protocols
**ACTIONS ON INTEGRATION OF NUTRITIONAL ASSISTANCE ACTIVITIES WITHIN THE TEAM (C) (25%)**	C1. Diet prescription in the medical records
	C2. Interconsultations on nutritional care
	C3. Team visits to patients
	C4. Participation in activities outside the HFNS
	C5. Nutritional support team
**ACTIONS SUPPORTING DIET THERAPY (D) (25%)**	D1.Diet manual
	D2.Information about energy supply
	D3.Selection of nutritional supplements
	D4.Mechanisms for patients to require changes to the diet
**FSQ INDICATORS (100%)**	**Employed criteria**
**MEDIATION ACTIONS WITH USERS AND OTHER HOSPITAL SECTORS (A) (25%)**	A1. Duty shift in the area of meal production
	A2. Formal evaluation of the HFNS regarding user satisfaction
	A3. Planning and goal-setting for the HFNS
	A4. HFNS participation in other hospital sectors
**AUTONOMY AND MANAGEMENT CONTROL ACTIONS (B) (25%)**	B1. HFNS responsibility for purchases
	B2. Budget autonomy
	B3. Control of cost/meal or cost/daily produced food
	B4. Statistical control by the HFNS
	B5. Statistical control of the produced diets
**MEAL PRODUCTION QUALIFICATION ACTIONS (C) (25%)**	C1. Standard prescription form
	C2. Dietetic kitchen
	C3. Routine tasting of diets
	C4. Good practice manual
	C5. Diet manual (*)
	C6. Production of nutritional supplements
**STAFF QUALIFICATION ACTIONS (D) (25%)**	D1. Staff evaluation
	D2. Instrument for staff evaluation
	D3. Periodic training program

(*) This criteria was considered important in both NCQ and FSQ.

The criteria were subdivided into 8 groups of actions, designated NCQ and FSQ indicators (4 groups of actions for each corpus). These indicators, consisting of between 3 and 6 criteria, were measured as a percentage of existence of that group of actions in a certain institution. Each indicator thus corresponded to 25% of the total NCQ and FSQ value. Food and Nutritional Care Quality in Hospital (FNCQH) was determined as the mean percentage that each institution complied with the NCQ and FSQ indicators, and comparisons in terms of the legal nature, type (general or specialized), size, and location of the hospital as well as the presence of a clinical dietitian were made. It was considered “clinical dietitian” the professional who works in the infirmary with the patient’s nutritional care. The existence of actions was assessed; however, the extent of their coverage was not evaluated.

**Table 2:** Characteristics of the hospitals

**Hospital Characteristics**	**Campinas**	**Ribeirão Preto**	**Florianópolis**	**Total**	
	**(n = 17)**	**(n = 10)**	**(n = 10)**	**(n = 37)**	**%**
Legal nature					
Public (n = 12)	3	2	7	12	32.4
Private (n = 25)	14	8	3	25	67.6
Total	17	10	10	37	100.0
Type					
Specialized	3*	2**	2***	7	18.9
General	14	8	8	30	81.1
Total	17	10	10	37	100.0
Size					
Small	2	1	3	6	16.2
Medium	11	6	4	20	54.1
Large	4	3	3	10	27.0
Total	17	10	10	37	100.0

* Maternity, oftalmologic and women hospital; ** maternities; ***maternity and infections and contagious disease hospital.

Descriptive statistical data analysis was also performed, and ANOVA models were employed for comparison of NCQ and FSQ among the studied institutions. Such model assumes that the residues obtained from the difference between values predicted by the model and the observed values have normal distribution with average 0 and constant variance. In situations in which this assumption was not confirmed, transformed-response variables were utilized [31]. Models were adjusted with the aid of the software SAS version 9.

## Results

Sixty-seven point six percent of the sample consisted of private hospitals, including philanthropic institutions (10 hospitals), whilst 32.4% were public hospitals, 18.9% of which were university hospitals and 13,5% were government institutions. In Florianópolis the majority of the sample was comprised of public hospitals (70,0%), whereas the cities of the state of São Paulo had a larger number of private institutions (81.5%) (Table 2). However, in terms of the public beds, Florianópolis had 79%; Ribeirão Preto had 67% and Campinas had 51%. Considering beds in specialized hospitals, we had 13,0% in Campinas (90,0% public beds), 7,3% in Ribeirão Preto (38,5% public beds) and 19,4% in Florianópolis (88,4% public beds). The investigated hospitals corresponded to 82.4% of the hospital beds available in the referred municipalities (Campinas 82.5%; Ribeirão Preto 87.5%; Florianópolis 76.0%), which represented a coverage of 5,566 beds [26]. There were 2 hospitals in Florianópolis, 2 in Ribeirão Preto and 2 in Campinas that did not accept to participate. There were 2 more hospitals in Florianópolis that were not included because one was being renovated and the other did not have a dietitian at that moment; and one in Campinas did not have a dietitian when we were collecting the data of the investigation.

As for HFNS human resources ratio, the average number of hospital beds per HFNS staff member and per dietitian was 3.81 (SD 1.98) and 68.04 (SD 43.26), respectively. Both indicators had a large coefficient of variation (Cv) (52.01% and 63.58%, respectively). Concerning meal production per staff member and per dietitian, the average number of produced meals was 20.55 (SD 14.61 and Cv 71.08%) and 320.65 (SD 173.67 and Cv 54.16%), respectively. There were a significant statistical difference between public and private hospitals to the number of hospital beds per HFNS staff member (p = 0.02) and per dietitian (p < 0.01) (Table 3). These variables were also significantly different for the hospitals located in Florianópolis, as compared to institutions situated in Campinas (p < 0.01) and Ribeirão Preto (p = 0.04).

**Table 3:** HFNS human resource indicators*

**Hospitals**	**n.b/HFNS sm**	**n.b/dt**	**n. HFNS sm /dt**	**n.meals/ HFNS sm**	**n.meals/ dt**
Legal nature					
Public hospitals (=12)					
Mean (sd)	3.26 ± (2.72)	36.32 ± (13.56)	14.01 ± (6.87)	25.4 ± (20.5)	297.67 ± (159.42)
Median	2.11	32.50	13.25	20.35	262.00
Coefficient of Variation	83.41	37.34	49.04	80.7	53.55
Minimum	1.72	16.54	3.20	11.4	98.00
Maximum	11.51	66.67	32.30	85.70	637.00
Private hospitals privados (=25)					
Mean (sd)	4.07 ± (1.51)	83.27 ± (44.48)	22.16 ± (13.28)	18.2 ± (10.5)	331,68 ± (182,22)
Median	4.02	70.00	21.00	15.83	308,00
Coefficient of Variation	37.07	53.42	59.94	57.8	54,94
Minimum	1.88	16.00	6.00	2.30	80,00
Maximum	9.33	169.00	60.00	50.0	780,00
Institution type					
Municipalities					
Campinas					
Mean (sd)	4.48 ± (2.47)	77.28 ± (46.62)	20.98 ± 15.01	19.28 ± (18.48)	281.82 ± (180.32)
Median	3.89	56.00	15.00	13.71	220.0
Coefficient of Variation	55.07	60.32	71.53	95.87	63.98
Minimum	2.25	30.00	3.20	2.33	80.0
Maximum	11.51	169.00	60.00	85.71	780.0
Ribeirão Preto					
Mean (sd)	3.82 ± (1.17)	84.29 ± (41,29)	22.18 ± (9.76)	18.21 ± (8.68)	363.5 ± (164,85)
Median	3.57	75.00	19.00	20.50	348.0
Coefficient of Variation	30.64	49.67	44.01	47.65	45.35
Minimum	2.11	25.00	11.90	6.25	115.0
Maximum	5.58	145.00	40.00	31.07	665.0
Florianópolis					
Mean (sd)	2.67 ± (1.11)	36.09 ± (16.76)	14.36 ± (7,13)	25.06 ± (11,81)	343.8 ± (173,74)
Median	2.08	32.50	14.00	22.39	332.00
Coefficient of Variation	41.75	46.44	49.64	47.11	50.53
Minimum	1.72	16.00	8.00	11.72	98.00
Maximum	4.63	66.67	32.30	50.00	637.00

* n.b/HFNS sm = number of beds per HFNS staff member; n.b/dt = number of beds per dietitian; n. HFNS sm /nt = number HFNS staff members per dietitian; n.meals/ HFNS sm = number of produced meals per HFNS staff member; n.meals/dt = number produced meals per dietitian.

Evaluation of the presence of actions related to criteria of the NCQ corpus revealed that the difference between public and private hospitals varied between 1.7% and 50.7%, and that the average of this difference were 17.4% (SD 14.9%). The actions with the most differences (≥30%) between public and private institutions were presence of routine visits to patients, evaluation of nutritional status, and existence of mechanisms for patients to require changes to the diet (Table 4), all of which predominated in the former type of health institution.

**Table 4:** NCQ criteria in hospitals

**NCQ criteria**	**Private Hospitals (n=25)**		**Public Hospitals (n=12)**		Difference*	**Campinas (n=17)**		**Ribeirão Preto (n=10)**		**Florianópolis (n=10)**	
	**n**	**%**	**n**	**%**	**%**	**n**	**%**	**n**	**%**	**n**	**%**
A - Inpatient dietary coverage actions											
A1. Duty shift system in the area of clinical nutrition	4	16.0	3	25.0	9.0	2	11.8	3	30.0	1	10.0
A2. Supervision of meal distribution in the ward	7	28.0	4	33.3	5.3	6	35.3	2	20.0	3	30.0
A3. Routine visits to patients	16	64.0	12	100.0	36.0	12	70.6	6	60.0	10	100.0
B – Evaluation and monitoring of nutritional status actions											
B1. Nutritional assessment (complete)**	4	16.0	8	66.7	50.7	7	41.2	1	10.0	4	40.0
B2. Nutritional status monitoring	8	32.0	6	50.0	18.0	7	41.2	4	40.0	3	30.0
B3. Entry of nutritional care information in the medical record	9	36.0	7	58.3	22.3	7	41.2	2	20.0	7	70.0
B4. Filling in forms about nutritional care	7	28.0	4	33.3	5.3	5	29.4	1	10.0	5	50.0
B5. Nutritional guidance at discharge	24	96.0	12	100.0	4.0	16	94.1	10	100.0	10	100.0
B6. Assistance protocols	9	36.0	6	50.0	14.0	10	58.8	3	30.0	2	20.0
C - Actions on integration of nutritional assistance activities within the team											
C1. Diet prescription in the medical records	15	60.0	7	58.3	1.7	12	70.6	4	40.0	6	60.0
C2. Interconsultations on nutritional care	7	28.0	5	41.7	13.7	5	29.4	3	30.0	4	40.0
C3. Team visits to patients	9	36.0	7	58.3	22.3	9	52.9	3	30.0	4	40.0
C4. Participation in activities outside the HFNS	18	72.0	9	75.0	3.0	11	64.7	9	90.0	7	70.0
C5. Nutritional support team	7	28.0	3	25.0	3.0	5	29.4	4	40.0	2	20.0
D – HFNS actions supporting diet therapy											
D1. Diet manual ***	16	64.0	4	33.3	30.7	14	82.4	3	30.0	3	30.0
D2. Information about energy supply	14	56.0	6	50.0	6.0	13	76.5	3	30.0	4	40.0
D3. Selection of nutritional supplements	7	28.0	4	33.3	5.3	5	29.4	4	40.0	2	20.0
D4. Mechanisms for patients to require changes to the diet	12	48.0	11	91.7	43.7	11	64.7	3	30.0	9	90.0

* Difference (in %) between Private and Public Hospitals; ** Nutritional assessment (complete) include patient’s history and anthropometric and biochemical data; *** This criteria was considered important in both NCQ and FSQ.

In all the studied cities, actions related to inpatient dietary coverage were rarely present in public and private hospitals (between 12% and 35% of the institutions developed these actions). A slightly better situation was found in terms of actions concerning assessment and nutritional status monitoring, which existed in 33.3% to 66.7% of the public hospitals. Nutritional guidance at discharge was one of the actions that were most present in both public and private institutions. As for the remaining actions, they occurred in approximately a third of the private hospitals.

When it comes to municipalities, hospitals in Florianópolis stood out particularly for entry of nutritional care information in the medical record and existence of a printed form specially designed for nutritional care.

As for actions related to integration of the health care team into nutritional care activities, over 70% of the HFNS of public and private hospitals participated in events held outside the institution, evidencing engagement of the service with nutritional issues. On the other hand, actions related to insertion of the dietitian into the health care team were more frequent in public hospitals; for instance, routine visits to inpatients and requirement for nutritional care interconsultation with the HFNS took place in public institutions more often.

Information about the diet energy supply, present in approximately half of the hospitals, and the choice of nutritional supplement offered by the HFNS, which occurred in approximately one third of the institutions, indicated that HFNS actions supporting dietotherapy must be implemented and valued, since they are essential for efficient delivery of inpatient nutritional care.

Evaluation of the criteria of the FSQ corpus revealed that the difference between public and private hospitals in terms of the presence of actions related to meal production was 17.3% (SD 13.5%) on average, varying between 1.0% and 39.3%. A higher number of private hospitals had a good practice manual, a diet manual, an instrument for staff evaluation, and a periodic training programme (Table 5).

**Table 5:** FSQ criteria in hospitals

**HMPQ criteria**	**Private Hospitals (n=25)**		**Public Hospitals (n=12)**		Difference*	**Campinas (n=17)**		**Ribeirão Preto (n=10)**		**Florianópolis (n=10)**	
	**n**	**%**	**n**	**%**	**%**	**n**	**%**	**n**	**%**	**n**	**%**
A - HFNS mediation actions with users and other hospital sectors											
A1. Duty shift in the area of meal production	7	28.0	3	25.0	3.0	5	29.4	5	50.0	0	0.0
A2. Formal evaluation of the HFNS regarding user satisfaction	7	28.0	3	25.0	3.0	6	35.3	3	30.0	1	10.0
A3. Planning and goal-setting for the HFNS	11	44.0	7	58.3	14.3	9	52.9	6	60.0	3	30.0
A4. HFNS participation in other hospital sectors	19	76.0	9	75.0	1.0	11	64.7	10	100.0	7	70.0
B - Autonomy and management control actions											
B1. HFNS responsibility for purchases	14	56.0	2	16.7	39.3	8	47.1	7	70.0	1	10.0
B2. Budget autonomy	5	20.0	1	8.3	11.7	4	23.5	2	20.0	0	0.0
B3. Control of cost/meal or cost/daily produced food	19	76.0	9	75.0	1.0	13	76.5	8	80.0	7	70.0
B4. Statistical control by the HFNS	21	84.0	12	100.0	16.0	14	82.4	9	90.0	10	100.0
B5. Statistical control of the produced diets	17	68.0	11	91.7	23.7	13	76.5	8	80.0	7	70.0
C - Meal production qualification actions											
C1. Standard prescription form	10	40.0	4	33.3	6.7	7	41.2	4	40.0	3	30.0
C2. Dietetic kitchen	13	52.0	8	66.7	14.7	8	47.1	8	80.0	5	50.0
C3. Routine tasting of diets	15	60.0	4	33.3	26.7	12	70.6	5	50.0	2	20.0
C4. Good practice manual	21	84.0	6	50.0	34.0	14	82.4	6	60.0	7	70.0
C5. Diet manual **	16	64.0	4	33.3	30.7	14	82.4	3	30.0	3	30.0
C6. Production of nutritional supplements	7	28.0	5	41.7	13.7	6	35.3	4	40.0	2	20.0
D – Staff qualification actions											
D1. Staff evaluation	13	52.0	2	16.7	35.3	5	29.4	5	50.0	4	40.0
D2. Instrument for staff evaluation	8	32.0	4	33.3	1.3	1	5.9	4	40.0	7	70.0
D3. Periodic training program	15	60.0	3	25.0	35.0	6	35.3	7	70.0	5	50.0

* Difference (in %) between Private and Pubic Hospitals; ** This criteria was considered important in both NCQ and FSQ.

Management control actions were strongly influenced by the legal nature of the institution, many of which reflected in the differences found between public and private hospitals. Among the actions that were detected in most of the institutions, HFNS participation in other hospital administrave spheres and cost control can be mentioned. In contrast, less than a third of the hospitals conducted evaluation regarding user satisfaction. It is noteworthy that less than half of the institutions had a standard diet prescription form (nutritional contents of food preparations furnished by the Service). There was a larger difference between public and private hospitals with respect to the meal production qualification actions. In the same way, it is important to note that few public institutions carried out staff qualification actions.

The mean percentage of compliance with the NCQ criteria in private and public hospitals was 41.6% (SD 19.8) and 51.8% (SD 16.1), respectively, and the median was 39.2% and 53.0%, respectively. As for FSQ criteria, compliance values were 49.1% (SD 20.7), median of 46.7% and 42.4% (SD 16.6), median of 42.7%, respectively.

Among the variables analyzed in Table 6, NCQ was positively influenced by hospital type (general) and presence of clinical dietitian. FSQ was affected by hospital size: medium-sized and large institutions were significantly better than small hospitals.

**Table 6:** Food and Nutritional Care Quality in Hospital measured as percentage of NCQ and FSQ

**Variables**	**INCQ (%)**						**HMPQ (%)**					
**Hospitals**	**Mean (SD)**	**Median**	**Coef Var**	**Minimum**	**Maximum**	**p**	**Mean (SD)**	**Median**	**Coef Var**	**Minimum**	**Maximum**	**p**
Legal Nature												
Public (n=12)	51.82 (16.07)	52.95	31.01	23.80	80.40	0.11	42.38 (16.55)	42.70	39.05	14.20	78.30	0.37
Private (n=25)	41.62 (19.84)	39.20	47.68	9.20	85.40		49.14 (20.69)	46.70	42.10	12.50	100.00	
Type												
General (n=30)	48.47 (17.79)	47.49	36.70	21.70	47.90	0.01	49.84 (19.78)	49.80	39.69	14.20	100.00	0.06
Specialized (n=7)	29.73 (18.09)	22.50	60.85	9.20	58.80		34.54 (12.78)	33.80	37.00	12.50	50.40	
Clinical dietitian												
yes (n=22)	39.11 (17.91)	37.70	45.80	9.20	85.40	0.02	50.15 (21.95)	48.55	43.78	12.50	100.00	0.32
no (n=15)	53.46 (18.05)	55.40	33.77	21.70	83.30		42.25 (14.59)	42.10	34.53	23.30	78.30	
Size												
Small (n=6)	31.82 (16.54)	30.85	51.97	9.20	52.90	a	29.4 (14.44)	29.80	49.10	12.50	46.30	b
Medium (n=21)	46.63 (18.65)	48.30	39.99	21.70	85.40		49.57 (18.57)	50.40	37.47	18.30	100.00	
Large (n=10)	49.21 (19.83)	51.05	40.29	21.70	80.40		51.96 (19.64)	49.80	37.80	30.00	91.70	
Municipality												
Campinas (n=17)	51.49 (20.06)	51.30	38.95	20.80	85.40	c	46.94 (19.15)	50.40	40.80	12.50	78.30	d
Ribeirão Preto (n=10)	36.05 (18.20)	31.05	50.50	9.20	62.90		57.01 (22.25)	48.55	39.03	31.70	100.00	
Florianópolis (n=10)	42.64 (15.43)	45.85	36.19	21.70	65.80		36.89 (12.05)	35.85	32.67	14.20	56.70	

a – no significant difference; b – significant difference between small and medium-sized hospitals (p=0.01) and between small and large hospitals (p=0.01) and no significant difference between medium-sized and large hospitals; c – significant difference between institutions in Campinas and Ribeirão Preto (p=0.04), no difference for the others; d – significant difference between Ribeirão Preto and Florianópolis (p=0.03), no difference for the others.

## Discussion

HFNS human resources, measured in terms of number of hospital beds and produced meals, are heterogeneous and characterized by a wide coefficient of variation, thus suggesting that there are no preset parameters for human resources ratio in this area.

The relation number of staff members per hospital bed, an indicator of hospital human resources in Brazil, varies between 1.0 and 7.2, while in public institutions this indicator varies from 4.0 to 9.0 [32]. Using this same relation for the number of HFNS staff members per hospital bed, an average of 0.27 (SD 0.09) and median of 0.25 is obtained. Differences found between human resources ratio in public and private hospitals with respect to the number of hospital beds per dietitian and the number of HFNS staff members per dietitian evidences a more favorable situation in public institutions in terms of graduated professionals. The difference among the investigated cities is probably a consequence of the larger concentration of public hospitals in Florianópolis. In a study on eight hospitals of four different Brazilian states [33] was observed a relation varying from 50 up to 150 patients per dietitian, associated with a situation of very precarious in same action analised about nutritional care.

Viabilization of nutritional care through evaluation of nutritional status and establishment of actions that suit their clinical and nutritional status along the duration of hospital stay depend on the relation number of hospital beds per dietitian [34]. Even if screening criteria are created and nutritional care levels are defined [35], values of 30 and 15 hospital beds per dietitian are recommended for general hospitals and more complex units, respectively [30]. The study that evaluated how nutritional risk is assessed and managed in European hospitals with twenty one thousand patients, found a range between 21% and 73% of the units from different regions that reported nutritional screening routine. The presence of dietitian, nutrition team and the screening nutrition routine increased the probability of to identifing nutritional risk [36].

More heterogeneous conditions were detected for the productivity indicator (number of produced meals per staff member). The number of meals produced for other segments such as hospital staff members and visitors may distort the aims of the HFNS and redirect human resources from the nutritional care sector to meal production. An increase in administrative demands may also deteriorate inpatient nutritional care actions [16,33].

Comparison between public and private health institutions in terms of NCQ criteria reveals that there are more actions related to nutritional care in public hospitals (routine visits to patients, evaluation of nutritional status, mechanisms for patients to require changes to the diet, entry of nutritional care information in the medical record, among others). Such actions may avoid or detect nutritional problems that can be handled during the hospital stay, thereby preventing nutritional aggravations [37]. This difference can be attributed to the presence of a larger number of clinical dietitian in public hospitals and to the fact that there are more university hospitals of public legal nature. The lack of nutritional assessment, diet prescription, and entry of nutritional information in medical records has also been detected in another work on Brazilian and Italian hospitals [16,33,38].

Some actions are lacking in both public and private institutions, with a difference smaller than 15% between them. Such lack of NCQ actions denotes little technical qualification for very important actions. Only half of the hospitals provide information about energy supply, whereas only a third has appropriate forms for registration of nutritional care, participation of the dietitian in meal distribution, and production of nutritional supplements by the HFNS. The legally prescribed nutritional support team [39] exists in a quarter of the hospitals only. These data reinforce the need for professional qualification and nutritional care action descriptors for the hospital segment [38].

Differences in FSQ criteria demonstrate that there is greater concern about staff evaluation and standardized procedures such as good practice and diet manuals in private institutions. Slight differences emerge when most of the NCQ and FSQ criteria are compared between public and private hospitals and among municipalities, which highlights the need for establishing general quality standards for an HFNS.

Among the FSQ actions, up to a third of the hospitals have duty shift in the area of meal production, carry out staff evaluation, and conduct formal evaluation of the HFNS regarding user satisfaction. There are actions that call for closer attention, since they take place in less than a third of the hospitals. For instance, despite being present in most of the public hospitals and in half of the private institutions, supervision of patient nutrition in the ward would very much facilitate establishment of mechanisms for patients to require changes to the diet if patient food ingestion was directly observed. Unfortunately, this contact between the dietitian and the patient has been overlooked [33,40]. As for differences concerning the responsibility of the HFNS for purchases, they are due to regulations regarding this procedure in public hospitals.

Request of nutritional care by physicians and other professionals by means of interconsultations demonstrates recognition of this type of action by the multiprofessional team. Nevertheless, this takes place in less than a third of private institutions. In public hospitals, the situation is slightly better, though below the desired frequency. Similar results indicating that food and nutritional care is still incipient in health institutions have also been reported by De Seta et al. [33].

Herein, the legal nature of the institution did not influence the food and nutritional care quality, in spite of the larger number of dietitian present in public hospitals. Evaluation of HFNS human resources ratio did not necessarily imply in better quality, since it also depends on professional qualifications and skills. Nevertheless, hospitals with clinical dietitians had substantially better NCQ, and 80% of the 15 institutions that hired a dietitian were public. The presence of a specialized professional in this area may make all the difference in terms of nutritional care quality not only because of qualification, but also because of the existence of institutional policies valuing these actions. However, it is noteworthy that an array of conditions should also exist, so that this type of care can be improved [37,41]. Professional qualification, institutional policies for the sector, nutritional care quality programmes such as accreditation and institutional evaluation [42], and sufficient number of professionals [34] can better qualify food and nutritional care in hospitals.

The more complex structure of general hospitals favors NCQ. A higher demand due to the diverse nutritional problems that may arise in general institutions may contribute to this result. Although there are no significant differences in relation to FSQ, data for general (49.8%) and specialized (34.5%) hospitals suggest that more complex units are more qualified in this area. Results concerning hospital size can be analyzed in the same light. Larger hospitals have higher FSQ. Additionally, size does not significantly influence NCQ, but scores rise with increasing hospital size. Furthermore, university hospitals may influence hospital nutritional care positively, once they are placed among general institutions. It should be borne in mind that five of the seven university hospitals included in this work are large.

The significant difference between the NCQ scores of hospitals located in two cities of the same state (both cities proportionally having the same number of university hospitals) and the lack of any difference as compared to institutions located in Florianópolis, where public hospitals are the majority, suggest that other factors operate when service qualification is being analyzed. Even if no differences among these municipalities exist in terms of staff qualification, differences regarding professional skills may be present.

## Conclusion

Food and nutritional care quality in hospital must be evaluated and qualified for improved efficacy of health institutions. The legal nature and size of hospitals may interfere in the examined segments, human resources score, NCQ, and FSQ. However, results from the present analysis lead to the conclusion that nutritional care is still incipient, and that actions concerning patient care and food service take place on an irregular basis in hospitals. This study allowed for attainment of a qualitative panorama of these actions and human resources indicators concerning hospital food and nutrition services.

The fact that coverage of the assessed actions was not verified is a limitation of the present work, which might have generated biased data. For instance, it is possible that some hospital units have excellent nutritional care teams while the care delivered to inpatients of some wards is deficient. The other limitation of the study was the frequency that these actions were developed. Both are important aspects that can influence in the quality of care. Some criteria influenced by the legal nature of the hospital may have given rise to some distortions.

Put together, our results show the performance of the actions developed by the HFNS. Although we cannot measure the coverage and the frequency of the actions, this study made a profile of the food and nutritional care in hospitals, an important step in guidelines for nutritional care in hospitals.

## Competing interests

The authors declare that they have no competing interests.

## Authors’ contributions

RWDG was the mentor of the work, was involved in the protocol and study design, data collecting and analysis, carried out the statistical analysis and wrote the manuscript. AS and RPCP participated in the planning of work, discussion and data collection in Florianópolis. VALM participated in the planning of work, discussion and data collection in Campinas. EZM made the statistical analysis and corrected the manuscript. All authors read and approved the final manuscript.
